# Anastomotic stenosis of the descending colon caused by barium granuloma formation following barium peritonitis: report of a case

**DOI:** 10.1007/s00595-013-0696-0

**Published:** 2013-08-19

**Authors:** Toshihiro Kitajima, Kenji Tomizawa, Yutaka Hanaoka, Shigeo Toda, Shuichiro Matoba, Hiroya Kuroyanagi, Yasunori Oota

**Affiliations:** 1 Department of Gastroenterological Surgery, Toranomon Hospital, Tokyo, Japan; 2 Department of Pathology, Toranomon Hospital, Tokyo, Japan

**Keywords:** Barium peritonitis, Barium granuloma, Anastomotic stenosis, Submucosa, Serosa

## Abstract

Anastomotic stricture reportedly often recurs following barium peritonitis, regardless of whether the anastomotic diameter is initially sufficient. However, the causes of repetitive stricture have not been clarified. We report a case that suggests the pathophysiology of recurrent anastomotic strictures following barium peritonitis. The patient was a 39-year-old Japanese man with idiopathic perforation of the descending colon after undergoing an upper gastrointestinal barium contrast study. After emergency peritoneal lavage and diverting colostomy, created using the perforated region, the patient recovered uneventfully and 3 months later, the colostomy was closed and the perforated colon was resected. However, 7 months after colostomy closure, abdominal distention gradually developed, and colonoscopy revealed an anastomotic stricture. The patient was referred to our hospital where he underwent resection of the anastomotic stricture. The surgical specimen exhibited barium granulomas not only in the subserosa of the entire specimen, but also in the submucosa and lamina propria localized in the anastomotic site. These findings suggest that barium was embedded in the submucosa and lamina propria with manipulation of the stapled anastomosis and that the barium trapped in the anastomotic site caused persistent inflammation, resulting in an anastomotic stricture.

## Introduction

Experimental studies on animal models have revealed that barium peritonitis has the distinct potential to induce severe inflammation [1, 2] and that the development of barium granulomas represents chronic change following barium peritonitis [3]. However, most reported cases of barium granulomas have occurred in the rectum as an unusual complication of barium enemas [4–6], whereas barium granulomas have rarely been detected in anastomotic sites following barium peritonitis. A previous report indicated that repetitive anastomotic stenosis following barium peritonitis is caused by barium granulomas adherent to the serosa of the colon [7]. However, the mechanisms underlying the development of such anastomotic strictures have not been clearly assessed. We report a case of anastomotic stenosis following barium peritonitis, showing distinctive pathological features indicative of the mechanisms of this stricture.

## Case report

A 39-year-old Japanese man was admitted to our hospital with abdominal distention. The patient had no history of digestive disorders including carcinomas, and his family history was non-contributory. About 1 year earlier, he had undergone an upper gastrointestinal barium contrast study for a health check-up and experienced acute abdominal pain the next morning. Based on the results of radiography and computed tomography, barium peritonitis from perforation of the descending colon was diagnosed. Emergency lavage drainage and colostomy was performed, using the perforated region, at another hospital. The colostomy was closed 3 months later, using functional end-to-end stapled anastomosis at a different hospital.

**Fig. 1:**
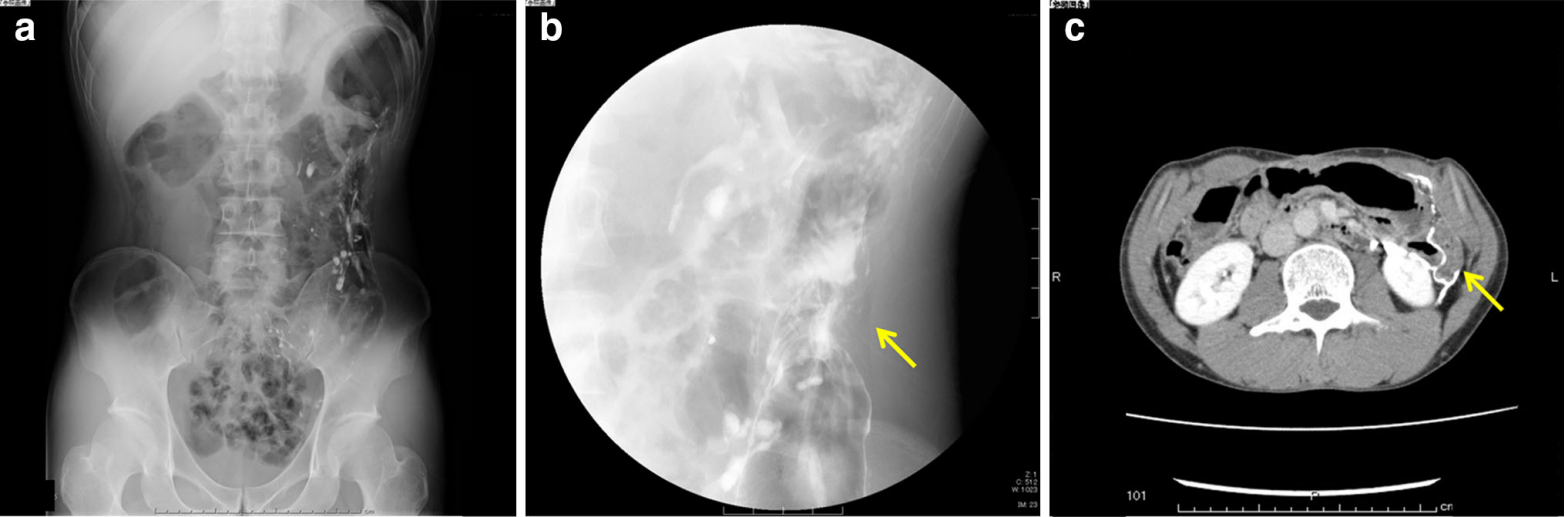
**a** Radiography, **b** gastrografin enema examination, and **c** computed tomography (CT) revealed stenosis of the descending colon (*arrows*) and remarkable distention on the oral side of the descending colon. Barium nodules were detected in the peritoneal cavity, especially near the anastomosis

However, the patient suffered from nausea and continually worsening abdominal distention, and colonoscopy revealed a pinhole-like stenosis at the anastomotic site. On admission to our hospital, radiography, gastrografin enema examination, and computed tomography revealed stenosis of the descending colon and remarkable distention of the oral side of the anastomosis (Fig. 1a–c). High-density areas suspected to contain barium were detected in the abdominal cavity, especially around the anastomotic site.

**Fig. 2:**
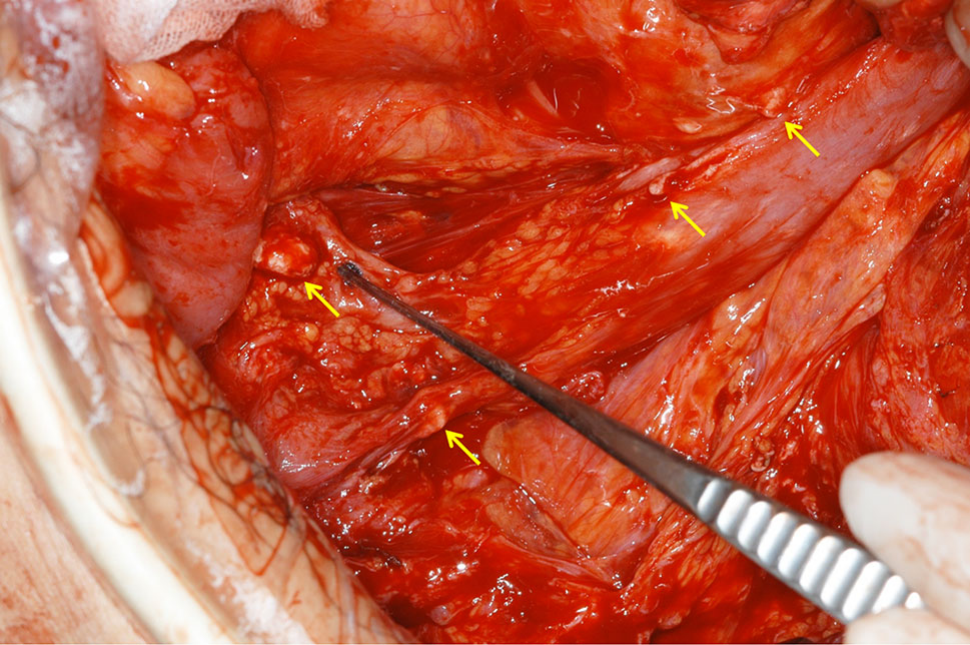
Several whitish nodules of barium were found in the abdominal cavity (*arrows*), firmly adhered to the bowel wall

**Fig. 3:**
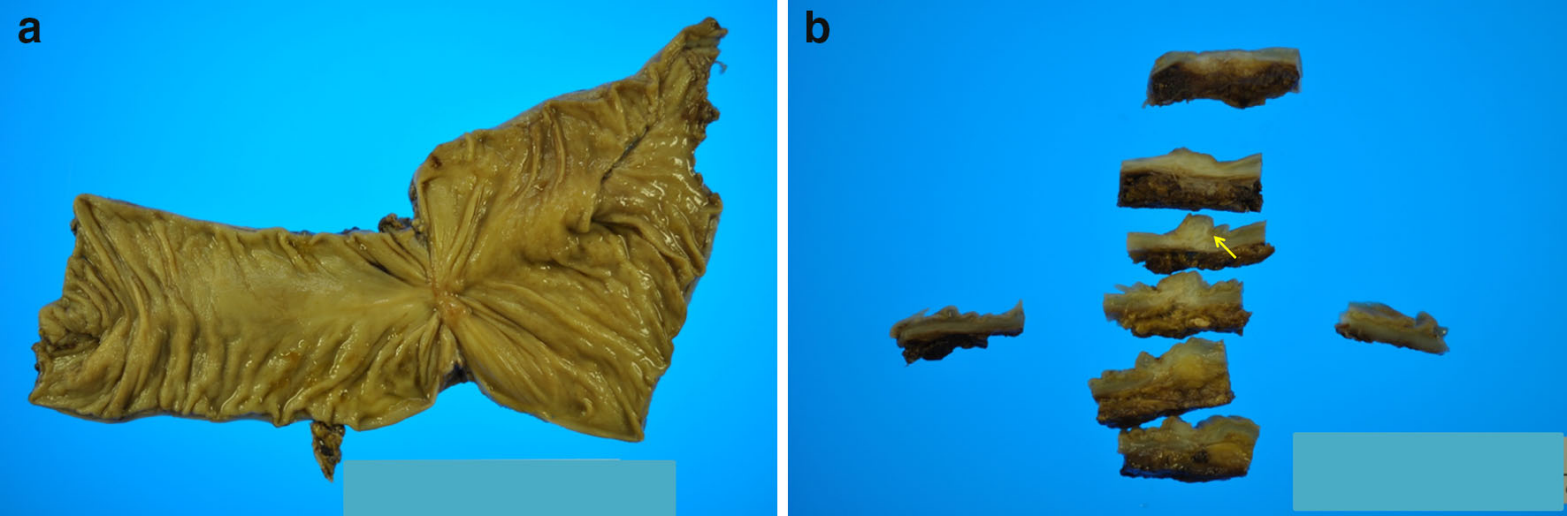
**a** The surgical specimen exhibited pinhole colon stenosis in the anastomosis. **b** Fibrosis was observed not only in the subserosa, but also in the submucosa and muscularis propria (*arrow*)

**Fig. 4:**
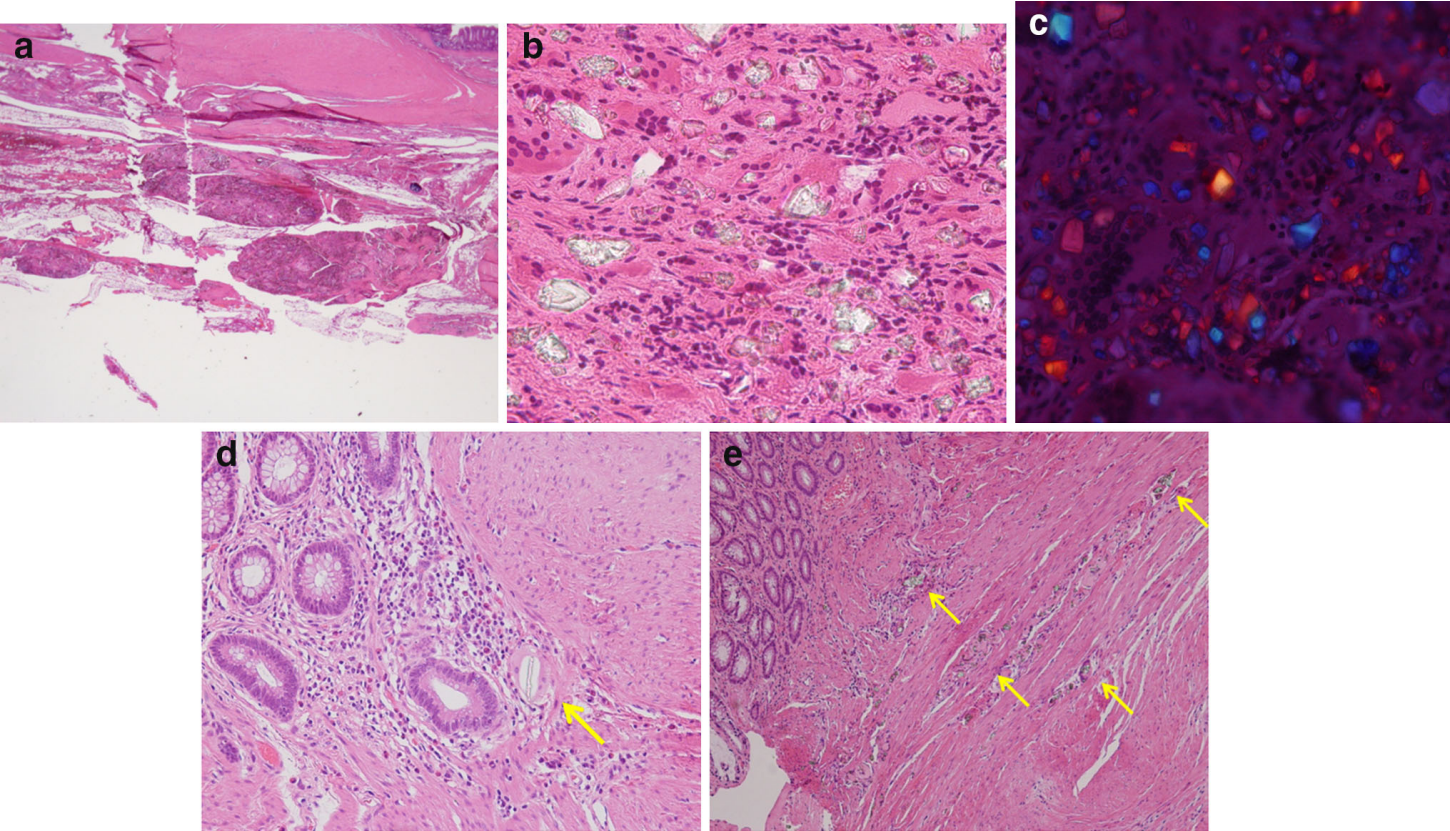
**a** Barium granulomas were present in the serosa. **b** Barium crystals were englobed by macrophages and multinucleated foreign body giant cells. This finding led to the diagnosis of barium granulomas. **c** The crystals were negatively birefringent, compatible with barium sulfate. **d**, **e** Barium granulomas were present in the submucosa and lamina propria, localized in the anastomosis (*arrows*)

The patient underwent emergency surgery to resect the anastomotic site, revealing severe adhesions between the bowel and several nodules including barium in the abdominal cavity (Fig. 2). The previous anastomotic site was located in the descending colon. Approximately 22 cm of colon was resected, followed by a functional end-to-end stapled anastomosis. When carrying out the anastomotic procedure, we confirmed that there were no areas of whitish maculation on the stump of the colon in each caliber. The surgical specimen contained pinhole anastomotic stenosis and whitish macular fibrosis in the submucosa and muscularis propria (Fig. 3a, b). Histologically, numerous granulomas around negatively birefringent crystals suggestive of barium were observed in the subserosa, even in the macroscopically normal sites (Fig. 4a–c). Furthermore, barium granulomas were detected in the submucosa and lamina propria only at the stricture site with marked fibrosis (Fig. 4d, e). Accordingly, a diagnosis of barium granulomas was made.

The patient recovered uneventfully and was discharged from our hospital on postoperative day 8. At the time of writing, 10 months later, no evidence of recurrence of anastomotic stenosis had been seen on colonoscopy.

## Discussion

Barium peritonitis is a rare and life-threatening complication of gastrointestinal contrast studies [8]. The incidence of peritonitis following barium enemas is reported as only 2–8 cases per 10,000 examinations [9]. Generalized peritonitis is extremely critical and difficult to treat because the rapid spread of barium over the peritoneal cavity can lead to the exudation of a large volume of fluid and albumin [8], which results in hypovolemia and fecal contamination with consequent sepsis [10]. In the present case, sufficient fluid resuscitation and the administration of antibiotics were effective in early removal of barium by peritoneal lavage during laparotomy.

A barium granuloma is defined as a granulomatous inflammatory lesion caused by the infusion of barium sulfate into the bowel wall [6]. According to previous reports, most barium granulomas of the gastrointestinal tract are detected in the rectum and caused by the insertion of contrast material in the rectal mucosa. This is primarily recognized as an uncommon complication of barium enemas [4–6]. The first documented case of barium granuloma of the rectum was reported in 1954 [11], since when there have been about 30 reports. Conversely, there are very few reports of barium granulomas of the colon. In 1993, Nishina et al. [7] reported a similar case in which the anastomosis stenosed twice following barium peritonitis and two-time partial colectomy. In that case, the strictured site was first anastomosed end-to-end; however, the treatment ended with colostomy, because of the repetitive stenosis. These authors concluded that the anastomosis was stenosed by barium granulomas firmly adhering to the serosa.

In our case, the microscopic findings revealed barium granulomas not only in the subserosa, but also in the submucosa and lamina propria (Fig. 4a–e). Notably, the granulomas in the submucosa and lamina propria were localized in the anastomotic site with marked fibrosis (Fig. 4d, e), which may have caused the anastomotic stenosis. We propose that barium was trapped in the submucosa and lamina propria with the stapled anastomosis, inducing repetitive, persistent chronic inflammation, which resulted in the anastomotic stricture. When performing anastomosis in patients with barium peritonitis, it is essential to confirm that there are no areas of whitish maculation on the stump of the colon in each caliber following sufficient lavage. In addition, the use of endoscopic follow-up is necessary to prevent recurrence of anastomotic strictures.

In conclusion, the microscopic findings of the present case indicated that the recurrent anastomotic stenosis following barium peritonitis was caused by the formation of barium granulomas in the submucosa or lamina propria, which had been entrapped in the anastomotic site. Thus, it is necessary to perform anastomosis in a way that prevents anastomotic stenosis. The compilation of a sufficient number of similar cases is warranted to generalize this finding.
